# Detecting antibodies to *Leishmania infantum* in horses from areas with different epizooticity levels of canine leishmaniosis and a retrospective revision of Italian data

**DOI:** 10.1186/s13071-020-04385-8

**Published:** 2020-10-22

**Authors:** Alessia Libera Gazzonis, Filippo Bertero, Iolanda Moretta, Giulia Morganti, Michele Mortarino, Luca Villa, Sergio Aurelio Zanzani, Benedetto Morandi, Riccardo Rinnovati, Fabrizio Vitale, Maria Teresa Manfredi, Luis Cardoso, Fabrizia Veronesi

**Affiliations:** 1 Department of Veterinary Medicine, Università Degli Studi Di Milano, Via dell’Università 6, 26900 Lodi, Italy; 2 Department of Veterinary Medicine, University of Perugia, Via S. Costanzo 4, 06126 Perugia, Italy; 3 Department of Veterinary Sciences, Alma Mater Studiorum, Università Degli Studi Di Bologna, Via Tolara di Sopra 50, 40064 Ozzano dell’Emilia, Bologna Italy; 4 National Reference Center for Leishmaniasis (C.Re.Na.L.), Istituto Zooprofilattico Sperimentale Della Sicilia, Via Gino Marinuzzi 3, 90129 Palermo, Italy; 5 Department of Veterinary Sciences, and Animal and Veterinary Research Centre, University of Trás-Os-Montes E Alto Douro (UTAD), Vila Real, Portugal

**Keywords:** Antibodies, Horses, IFAT, Italy, *Leishmania infantum*, Risk factors

## Abstract

**Background:**

*Leishmania infantum* is a vector-borne pathogen endemic in countries in the Mediterranean basin, including Italy. Dogs act as the primary reservoir for this parasite, but other animal species may also be infected. Low-to-moderate seroprevalence levels of infection have been reported in apparent healthy equine populations in southern Europe, reinforcing the importance of exploring those species, including horses, that act as a food source for vectors and may thus participate in the epizoological scenario of canine leishmaniosis (CanL) and zoonotic visceral leishmaniosis (ZVL). Since little is known regarding the exposure to *L. infantum* in horses in Italy, we assessed the seroprevalence in healthy equine populations from different CanL endemic areas.

**Methods:**

The survey was conducted on 660 apparently healthy horses distributed throughout central and northern regions of Italy between 2016 and 2019. Blood samples were collected and the presence of anti-*Leishmania* antibodies (IgG) was investigated by the immunofluorescence antibody test. Information on the location and altitude of the stables, along with the horses’ breed, age, sex, and reproductive status was obtained by filling in a questionnaire. This was then used for statistical analysis by generalized linear models to explore risk factors associated with seroreactivity to *L. infantum*.

**Results:**

An average seroprevalence of 13.9% was detected for *L. infantum* in the equine populations investigated, with statistically significant associations between seroprevalence, geographical variables (northern *vs* central Italy, origin and altitude) and individual factors (i.e. age and breed morphotype).

**Conclusions:**

Our results highlight that horses are frequently exposed to *L. infantum*. Further prevalence surveys in horses, also using direct methods (e.g. PCR), are warranted to clarify the role of these hosts in the epidemiology of *Leishmania* in Italy.
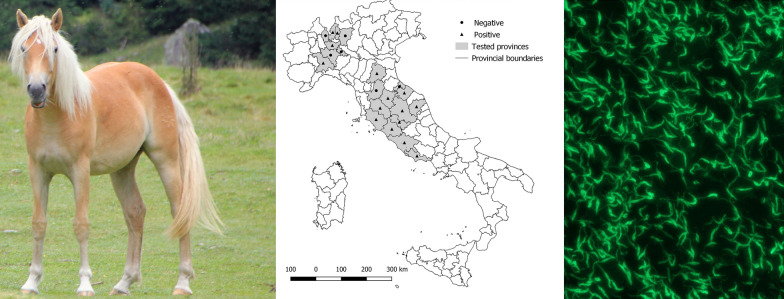

## Background

The vector-borne parasitic protozoan *Leishmania infantum* is endemic in countries of the Mediterranean basin, including Italy where our study was carried out [1–3]. Dogs act as the primary or main reservoir of the parasite; however, other animal species may also become infected, in particular carnivores (e.g. cats, foxes, wolves, mustelids and viverrids) and rodents, hares, cattle and horses [4–7].

Horses have received less attention than other *Leishmania* hosts in view of the overall low numbers of clinical cases reported, the mild clinical picture without visceral involvement, and the self-recovery of cutaneous lesions [8, 9].

Most descriptions of equine leishmaniosis (EL) have been caused by *Leishmania braziliensis* and have been recorded in equids from South and Central America [10, 11], where horses and donkeys are suspected to be a reservoir for human cutaneous leishmaniosis [12, 13].

However, in the past 20 years, confirmed clinical cases of EL, almost all them caused by *L. infantum*, have been reported in traditionally canine leishmaniosis (CanL) endemic areas of southern Europe (i.e. Spain and Portugal), and also in non-endemic areas of central Europe (i.e. Germany and Switzerland) at the border of the northern limit of CanL distribution [4, 8, 9, 14–17]. To the best of our knowledge, in Italy no report of clinical EL has been described to date.

Low-to-moderate seroprevalence levels of infection have been reported in apparently healthy equine populations of southern Europe, i.e. Portugal, Italy, Greece and Spain [16, 18–21], reinforcing the importance of exploring how horses contribute to the epizoological scenario of CanL and zoonotic visceral leishmaniosis (ZVL) [22].

Horses may contribute to the maintenance of the vector populations [23], acting as an excellent attraction and blood supply for the adult females of sand flies [24], with their faeces being an important source of food for sand fly larval development [25]. Moreover, a study carried out in an emerging area of high endemicity for visceral leishmaniosis in South America showed that the presence of horses increased the risk of *Leishmania* spp. epizootic in domestic dogs [26].

Since little is known about the exposure to *L. infantum* in horses, the main goal of the present study was to assess the seroprevalence in a healthy horse population in Italy. In addition, to understand the role of horses in the epidemiology of *Leishmania* in various areas of the country with different degrees of CanL epizootic spread, further aims were to evaluate: (i) individual risk factors associated with seroprevalence; and (ii) the potential role of horses as an indicator of the spread of *L. infantum*.

## Methods

### Study areas and population

**Fig. 1:**
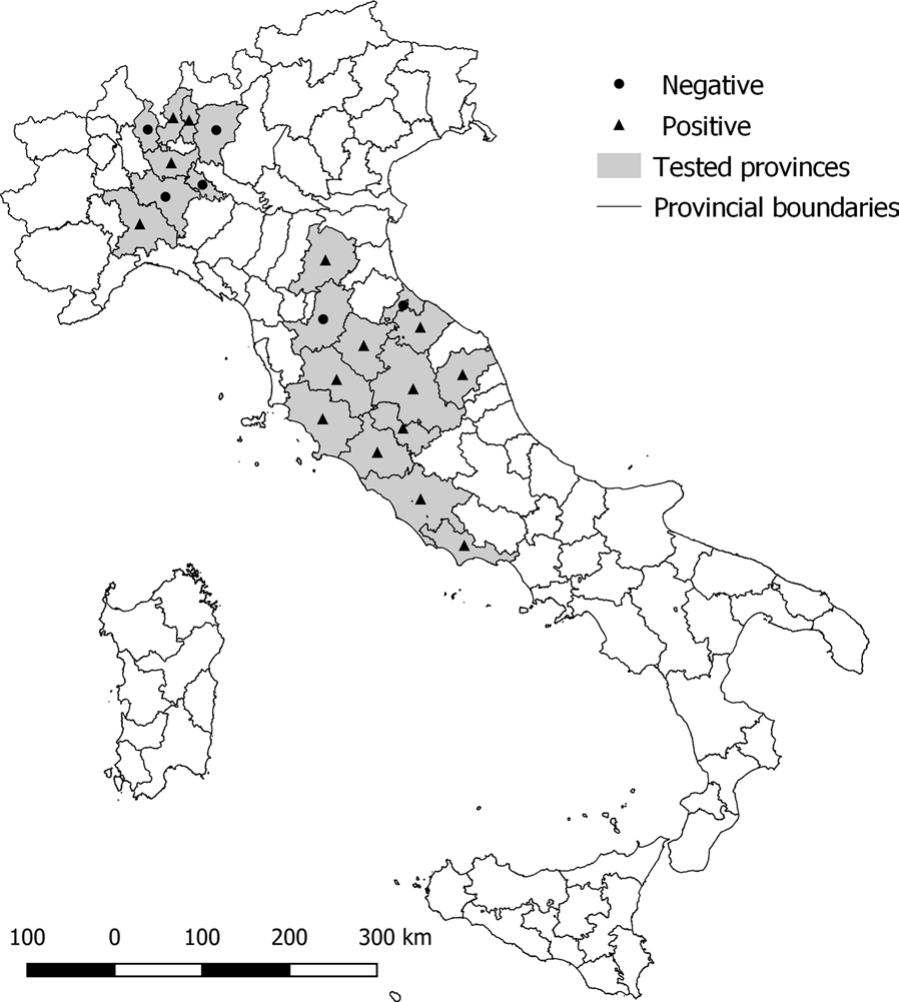
Sampling and positive areas to *Leishmania infantum* in horses from each site across the Italian peninsula

The study population consisted of 660 apparently healthy horses from two macro-areas, i.e. three regions in northern Italy (Lombardy, Piedmont and Emilia Romagna) and four regions in central Italy (Lazio, Tuscany, Marche and Umbria), reported as having a different pattern of epizooticity for CanL (Fig. 1) [27].

The sampling size for the two macro-areas was obtained considering an overall number of horses reared of 109,358 and 94,307 in the northern and central regions, respectively [28], together with a 50% expected prevalence with a 95% confidence interval (CI), and an absolute error of 5.7% and 5.13% for the northern and central regions, respectively. Between October 2016 and October 2019, a convenience blood sample was taken from apparently healthy horses from the external jugular vein. Each sample was centrifugated at 3000 × *rpm* for 10 min. The serum was then separated and stored at − 20 °C until used for the serological assay.

An epidemiological questionnaire was administered to the owners during the blood sampling in order to collect data on putative risk factors for seropositivity to *L. infantum*, including sex, age, and breed. In addition, the location and the altitude of the municipalities of the stables where the horses were kept were recorded (https://www.istat.it/it/archivio/156224). Apart from ponies, breeds were classified based on their morphological types [29] as mesomorphic (i.e. Arabian and Haflinger), meso-brachymorphic (i.e. Friesian), meso-dolichomorphic (i.e. Italian Saddle, Quarter Horse and Andalusian), or dolichomorphic (i.e. trotters and thoroughbred), as reported in Table 1.

**Table 1:** Horses from two macro-areas in Italy included in the study according to morphotype and breed

Morphotype	Breed	*n*
Dolichomorphic	Anglo-Arabian	2
	French trotter	2
	Italian trotter	1
	Quarab	2
	Standardbred	56
	Thoroughbred	76
Mesomorphic	Appaloosa	3
	Arabian	38
	Bardigiano	4
	Criollo	19
	Haflinger	16
	Irish Hunter	1
	Lipizzan	1
	Mérens	1
	Nonius	1
	Tolfetano	54
	Wielkopolski	2
Meso-brachymorphic	Friesian	4
	Irish Cob	2
Meso-dolichomorphic	Andalusian	12
	Belgian warmblood	4
	Budyonny	1
	Dutch warmblood	17
	Hanoverian	3
	Italian Saddle	114
	Maremmano	7
	Oldenburg	1
	Paint Horse	10
	Quarter Horse	54
	Selle Français	3
	Swedish Warmblood	1
	Ukrainian Riding Horse	1
	Warmblut	1

*Abbreviation*: n, number of horses

### Serological analysis

The presence of anti-*Leishmania* antibodies (IgG) was investigated by an immunofluorescence antibody test (IFAT), following the standard procedures recommended by the Office International des Epizooties (OIE, World Animal Health Organization) [30]. Promastigotes of *L. infantum* zymodeme MON-1 (MHOM/TN/80/IPT-1) were used as the antigen, while rabbit-anti-horse -IgG-FITC (F7759; Sigma-Aldrich Chemical, Darmstadt, Germany) diluted at 1:30 was used as the conjugate. Serial dilutions were performed. The samples that showed reaction in dilutions equal or higher than 1:40 were considered positive. Since a standardized cut-off is not available for the detection of anti-*L. infantum* antibodies in horses, the 1:40 cut-off was used assuming that it is indicative of exposure but not necessarily of an established infection as in the case of CanL [30]; moreover, it is the most used cut-off in the case of epidemiological screening for *Leishmania* in equine populations, allowing the comparison of the results obtained [31]. End-point titers of the positive serum samples were determined. The positive control for *Leishmania* consisted of sera from Portugal, obtained from a horse presenting active leishmanial lesions, which tested positive by the direct agglutination test (DAT), with a titre of 800 (cut-off: 200) and PCR. The negative control consisted of an animal that had previously tested negative by both DAT (titre < 25) and molecular assays.

### Statistical analysis

The seroprevalence at an individual level was computed with the associated 95% confidence interval (95% CI). For the descriptive statistics, age and altitude, computed in months and meters above sea level (m.a.s.l.) respectively, were categorized as specified below. For males, a possible age difference between geldings and stallions was verified using the Student’s t-test. Univariable general linear models (GLMs) with a binomial distribution and logit link function were performed to explore the relation between seroreactivity to *L. infantum* and associated risk factors, including origin (northern and central Italy), altitude (< 200 m.a.s.l.; 200–500 m.a.s.l.; and > 500 m.a.s.l.), age (continuous variable in years), sex (female and male), reproductive status of males (gelding and stallion), and breed morphotype (mesomorphic; meso-brachymorphic; meso-dolichomorphic; and dolichomorphic). All variables and their two-way interactions were subsequently entered into a multivariable model, developed by backward elimination until all the remaining variables were significant (*P*-value < 0.05) or not. The estimated means were then compared through pairwise comparisons. The goodness-of-fit of the model was assessed by the Akaikeʼs information criterion (AIC). Results were presented as adjusted odds ratios (OR) with 95% CI. Statistical analysis was performed using commercial software (SPSS, Version 22.0; Chicago, IL, USA).

## Results

Serological results showed that 92 out of the 660 examined horses (13.9%, 95% CI: 11.4–16.8%) tested positive for anti-*L. infantum* antibodies. Most of the samples (*n* = 70) were positive at a 1:40 dilution/cut-off; 21 samples were positive at a 1:80 dilution and only one sample at a 1:160 dilution.

A higher seroprevalence was recorded among horses that tested seropositive for *Leishmania* from the central regions (15.3%) compared to those from northern Italy (12.2%), although the difference was not statistically significant (*P*-value = 0.248) (Table 2). The exact location of the stables and thus the altitude of the municipalities where the stables were located was recorded for 464 animals. Altitude was significantly associated with seropositivity (*P*-value = 0.025): most positive animals were kept in stables located at an altitude of between 200 and 500 m.a.s.l. (i.e. 31 out of 172 examined, 18%), while a lower number of seroreactive animals was recorded in animals living at altitudes below 200 (i.e. 18 out of 147 examined, 12.2%), or above 500 m.a.s.l. (i.e. 11 out of 145 examined, 7.6%).

**Table 2:** Distribution of frequencies and risk factors associated with the presence of anti-*L. infantum* antibodies by IFAT in sampled horses (*n* = 660) according to the univariate analysis by the generalized linear model

Variable	Category	Positive/examined	%	Wald’s Chi-square	β ± SE	OR	95% CI	*P*-value
Origin	Central Italy	56/365	15.3	1.335	0.265 ± 0.2296	1.304	0.831–2.045	0.248
	Northern Italy	36/295	12.2		0	1		
Altitude (m.a.s.l^.^)				63.532				0.025
	< 200	18/147	12.2		0.531 ± 0.4021	1.7	0.773–3.738	0.187
	200–500	31/172	18.0		0.985 ± 0.3711	2.678	1.294–5.543	0.008
	> 500	11/145	7.6		0	1		
Age	Continuous			5.243	0.040 ± 0.0175	1.041	1.006–1.077	0.022
Sex	Female	32/287	11.1	3.280	− 0.426 ± 0.2353	0.653	0.412–1.036	0.070
	Male	59/366	16.1		0	1		
Reproductive status (only males)	Geldings	46/245	18.8	3.770	0.653 ± 0.3361	1.920	0.994–3.711	0.052
	Stallions	13/121	10.7		0	1		
Breed morphotype				60.020				0.003
	Mesomorphic	17/140	12.1		0.691 ± 0.4310	1.996	0.858–4.646	0.109
	Meso-dolichomorphic	44/229	19.2		1.234 ± 0.3833	3.435	1.621–7.282	0.001
	Meso-brachymorphic	0/6	0.0		–	–	–	–
	Dolichomorphic	9/139	6.5		0	1		
Horse *vs* ponies	Horses	74	12.4	8.175	0	1		
	Ponies	9	32.1		1.210 ± 4.233	3.354	1.463–7.689	0.004

*Abbreviations*: m.a.s.l., meters above sea level; β ± SE, coefficient ± standard error; OR, odds ratio; CI, confidence interval

The age of 599 horses was known (mean ± standard deviation (SD): 9.77 ± 6.3; min–max: 0–35 years). The age was found to be strongly associated with seropositivity, with a risk of infection rising with the increase in age (OR = 1.041, *P* = 0.022) (Table 2). Eleven out of the 125 young horses (≤ 4 years) included in the study tested positive (8.8%), while of the adult horses, 58 out of the 352 animals aged between 4 and 15 years (16.5%) and 21 out of the 122 older than 15 years (17.2%) were shown to be seropositive.

Thirty-two females (11.1%) and 59 males (16.1%) tested positive; the sex of 7 horses was not known. No difference was recorded between males and females (*P* = 0.070). Considering only males, a slight difference, at the limits of significance, was recorded between geldings (46/245, 18.8%) and stallions (13/121, 10.7%) (*P* = 0.052) (Table 2). A difference for age was recorded between geldings (mean ± SD: 11.17 ± 5.92 years) and stallions (mean ± SD: 7.58 ± 5.79 years) (*t* = − 5.311, *df* = 331, *P* = 0.0001).

Data on breeds were obtained for 626 animals: 514 horses belonged to one of the 33 breeds represented in the study; in addition, 84 cross-breeds and 28 ponies were sampled (Table 1). A higher prevalence was obtained in ponies (32.1%) than in comparison (12.4%) (*P* = 0.004) (Table 2). The morphotype of purebred horses was found to be a predictive factor of infection: dolichomorphic horses showed the lowest seroprevalence (6.5%) compared to mesomorphic (12.1%) and meso-dolichomorphic animals (19.2%). In particular, meso-dolichomorphic horses were statistically more at risk of being seroreactive than dolicomorphic horses (*P* = 0.001) (Table 2). Meso-brachymorphic animals were not considered in the analysis, since all the six horses included in this group were seronegative. In addition, four cross-breed horses (4.8%) were seropositive.

The same variables and their two-way interactions were then entered into a multivariable model. Two final models were considered. Considering individual variables (age, sex, and breed morphotype), only breed morphotype was retained in the final model (AIC = 19.91). A second multivariate model (AIC = 34.79) included the origin, altitude, and their interaction (Table 3). In fact, a different pattern was depicted in the distribution of serorevalence between northern and central Italy: the highest values were recorded among 200 and 550 m.a.s.l. in the north (22/97, 22.7%), and below 200 m.a.s.l. in central Italy (6/36, 16.7%).

**Table 3:** Distribution of frequencies and risk factors associated with the presence of anti-*L. infantum* antibodies by IFAT in sampled horses according to the multivariable generalized linear model

Variable	Category	Positive/examined	%*	Wald’s Chi-square	β ± SE	OR	95% CI	P-value
Origin				2.201				0.138
	Central Italy	56/365	15.3	5.249	1.835 ± 0.8011	6.268	1.304–30.132	0.022
	Northern Italy	36/295	12.2		0	1		
Altitude (m.a.s.l.)				6.248				0.044
	< 200	18/147	12.2	3.980	1.553 ± 0.7786	4.727	1.028–21.746	0.046
	200–500	31/172	18	10.391	2.437 ± 0.7560	11.440	2.599–50.347	0.001
	> 500	11/145	7.6		0	1		
Origin × altitude (m.a.s.l.)				9.257				0.010
	Central Italy × < 200	6/36	16.7^ab^	1.905	− 1.335 ± 0.9671	0.263	0.040–1.752	0.168
	Central Italy × 200–500	9/75	12.0^b^	8.185	− 2.601 ± 0.9093	0.074	0.012–0.441	0.004
	Central Italy × > 500	9/65	13.8^b^		0	1		
	Northern Italy × < 200	12/111	10.8^a^		0	1		
	Northern Italy × 200–500	22/97	22.7^b^		0	1		
	Northern Italy × > 500	2/80	2.5		0	1		

^*^Values of seropositivity per range of altitude with different superscript letters (a, b) are statistically different from each other. P-value < 0.05, GLM, pairwise comparison

*Abbreviations*: m.a.s.l., meters above sea level; β ± SE, coefficient ± standard error; OR, odds ratio; CI, confidence interval

## Discussion

We investigated the exposure to *L. infantum* in apparently healthy horses from two of the main macro-areas of Italy (i.e. northern and central Italy). An overall seroprevalence of 13.9% was recorded. Of the 92 detected seropositivities, the antibody titers were quite low with 70 samples testing positive at the cut-off dilution (1:40), which could be considered as an expression of a past contact with the parasite but without evidence of a current active infection. The detection of *Leishmania* spp. DNA in the blood samples or in lesions of infected horses as observed in previous surveys [14, 24, 32] might have supported evidence of active infection in horses but was not conducted.

The prevalence levels recorded are higher than those reported in similar endemic countries of the southern Europe, such as Greece and Portugal, which ranged from 0.3% to 4%, respectively [16, 19].

At a national level, the seroprevalence observed in both macro-areas investigated was higher than previously detected in apparently healthy horses (6.4%) [20] from central Italy (Tuscany), but lower than that recorded in the same area in donkeys (36.7%) [21]. Considering horses from the same area (Tuscany), in the present study the seroprevalence obtained was 11.7% (6/51) and even lower considering only sporting horses (2/39, 5.1%). The difference in seroprevalence values between horses, especially sporting horses, and donkeys can be attributed to the differences in their management. In fact, donkeys are more often kept outside compared to sporting horses, which are managed indoors, as already suggested for other protozoans that affect equids [33–36]. In addition, although not yet demonstrated, horses may have a greater susceptibility to infection, or alternatively donkeys may have a more efficient response of the immune system to the antigenic stimulus.

Differences in the seroprevalence values reported in the literature may also depend on the diagnostic techniques used. In fact, there is a strong lack of homogeneity in data obtained from different studies as regards the serological techniques (mainly IFAT and enzyme-linked immunosorbent assay [ELISA]) and the cut-offs used. In our study, the exposure of equids to *L. infantum* was assessed using IFAT, as previously described for other *Leishmania* hosts (e.g. humans, dogs and cats) [37, 38] and according to similar studies performed on horses in South America and Europe [31, 39]. In horses, an IFAT cut-off titer for *Leishmania* has not yet been defined, however a 1:40 dilution cut-off has been suggested as an adequate indicator of a specific reaction [31, 40].

The results obtained in central and northern Italy showed variable seroprevalence levels probably depending on the epizooticity of the areas and thus on the infective environmental pressure. In northern Italy, which is considered to have a medium-to-low epizooticity for CanL [2, 27], the prevalence of antibodies to *Leishmania* found in the equine populations was lower (12.2%) than the homologous seroprevalence values found in horses from central Italy (15.3%), which is regarded as having medium-to-high and stable epizootic areas for CanL [2, 15, 27, 41].

As a result of environmental changes and socio-economic factors, the distribution area of *L. infantum* in Italy, and more generally in Europe, is expanding northwards, with the expansion of endemic areas and with the appearance of new foci of infection in non-endemic areas [42]. In fact, climate change and the movement of reservoir hosts from endemic to non-endemic or hypo-endemic areas has led to the expansion of the distribution area of sand flies and *L. infantum* [43]. Although the recorded seroprevalence values did not significantly differ between northern and central Italy, statistical analysis showed a different association between the seroprevalence and the altitude of the locality of the stable in which the horses were housed between the two macro-areas. In fact, there was a different distribution of cases of infection among horses: in the northern regions, most of the seroreactive animals (22/97, 22.7%) came from stables located at an altitude of between 200 and 500 m.a.s.l., with a prevalence, corresponding to the optimal altitude range for vector host sand flies [44]. However, in the central regions, the seroreactivities were uniformly distributed, with the greatest number of positive samples from locations at an altitude of less than 200 m.a.s.l. (6/36, 16.7%).

Environmental variables (i.e. climate, location, and human population density) were also taken into consideration in a previous study, without however determining an association with the distribution of cases of positivity to *L. infantum* in horses [45].

The differences in seroprevalence depending on the altitude in the two considered macro-areas found in our study by the multivariate final GLM could be attributed to a different distribution of vectors within Italy [42], due to a different territory orography that may modulate the spread both of vectors and of *L. infantum* infection in the equine population. *Phlebotomus perniciosus* represents the most common sand fly species in the studied areas [44, 46, 47], and the seroreactivity detected in the investigated equine populations suggests that given that *P. perniciosus* does not have a host feeding preference it may include horses among its sources of food. To support this, an investigation conducted in central Italy to identify blood-meal sources of sand flies showed that *P. perniciosus*, *Phlebotomus perfiliewi*, *Phlebotomus papatasi* and *Phlebotomus mascittii* do feed on horses [44].

Similarly, the presence and abundance of primary and secondary hosts may impact on the parasitic pressure in the environment and therefore the exposure to *L. infantum* may vary depending on where the horse is housed.

Moreover, since horses often move from urban to semi-urban areas having different degree of biodiversity, they may be subjected to a different parasitic pressure depending on the habitat and therefore to a different risk of acquiring *L. infantum* infection [48].

Although comparisons among prevalence data obtained at the national level are difficult, since a wide variety of diagnostic approaches were used (e.g. PCR, serology by IFAT, classic or rapid-ELISA and western blotting), data concerning the spread of *L. infantum* infection in domestic and wild animals in Italy were considered (Additional file 1: Table S1). The seroprevalence values assessed in domestic animals ranged between 2.5–40.3% in dogs and 1.3–11.2% in cats [27, 49–57], which could be considered in line with those detected in the investigated horse populations in the present study (13.9%). Considering wildlife (i.e. hares, foxes, wolves and rodents), most of the surveys carried out in central and northern Italy have been conducted using molecular tools and showed levels of positivity from low in hares sampled in Tuscany (0.9% by IFAT and 9.8% by PCR) [58, 59] and in black rats (15.5%) [60] to high in red foxes (52.2%) in central Italy [61] and badgers (53.3%) in northern Italy [62].

Apart from the level of epizooticity of the study area and the co-presence of dogs and other hosts, little is known about the other risk factors involved in *L. infantum* infection in equids. Few studies have in fact considered variables that could potentially modulate the spread of infection among horses (i.e. age, sex, activity and type of housing), however without defining risk factors statistically associated with the infection [16, 45, 63]. The univariate analysis by GLM demonstrate an increase in the risk to be seropositive to *L. infantum* with the increase of age. Although this finding does not match other data recorded in horses [16, 45, 63], the risk of infection is assumed to increase with increasing age due to horizontal transmission by vectors as well as the situation observed in dogs in which age represents a well-defined risk factor for *L. infantum* infection [64, 65].

No difference was recorded between males and females; besides, the higher numbers of seroreactivity found in geldings compared to stallions is probably attributable to age rather than to factors directly related to the reproductive status of the animal.

Lastly, possible differences related to breeds were also considered. Ponies showed a higher seroprevalence than horses, as confirmed by the univariate analysis by GLM, in agreement with a previous study [63]. However, the data prevent the speculation of a greater susceptibility of the host species, since other differences between horses and ponies, including their management, may be involved.

Regarding horses, a further difference in terms of seropositivity was recorded among the different morphotypes to which the breeds included in the study belonged, with the lowest level of seropositivity was detected among dolichomorphic breeds. These differences, confirmed as statistically significant by the final multivariate GLM on individual variables, could be due to a different use of the horses by owners depending on the breed. In fact, the dolichomorphic breeds included in our study consisted of trotters and Thoroughbred horses, which have a marked aptitude for equestrian sports (particularly show jumping and racing) and thus their management involves being kept indoors with access to the paddock only for training or competitions. On the other hand, breeds belonging to the other morphotypes (i.e. Haflinger among the mesomorphic, and Quarter Horses among the meso-dolichomorphic) are more often used for non-competitive riding or for recreational purposes (i.e. trekking or riding courses for children) and their housing is more often mixed (indoors/outdoors) and they are kept outside for longer than competition horses. Our findings are in line with other authors [16] who found seropositivity to *L. infantum* only among horses used for recreational purposes, and not in farming or sporting horses. Therefore, it can be indirectly assumed that the horse use and its management type could be involved in the altered spread of *L. infantum* infection, as already described for other vector-borne infections [66].

In our study, none of the seropositive horses showed clinical signs compatible with leishmaniosis. Since no complementary clinicopathological analyses were conducted, visceral signs cannot be excluded, as well as possible temporary cutaneous lesions. Cutaneous leishmaniasis is the only clinical feature described in horses [4, 8]. The pathogen produces a variety of cutaneous lesions including single or multiple nodules of variable size, or papular lesions on the head, limbs or axillary and inguinal regions, without visceral signs [4, 8, 17].

However, seropositive results in healthy horses may be common in endemic areas, and this has already been observed for instance in Spain and Portugal [16, 18]. This is also comparable to findings in dogs and humans infected by *L. infantum*, which, often mount a specific immune response without developing clinical signs [67]. As previously observed in dogs, cats and humans, in areas of endemicity, the prevalence of subclinical *Leishmania* infection in horses is considerably higher than that of the disease itself. The absence of evident cutaneous and/or visceral signs may be related to a possible immunological ability to reduce the parasitic load or to what may be only temporary signs that are difficult to detect [24].

## Conclusions

The results of this study provide valuable information regarding the exposure to *L. infantum* among horses in central and northern regions of Italy. In addition, they highlight the importance of further investigating (e.g. by using molecular tools) the role of horses in the complex epidemiological cycle of leishmaniosis in these epizootic areas.

## Supplementary information

**Additional file 1: Table S1.** Comparison of prevalence of *L. infantum* infection among wildlife and domestic animals from two macro-areas (central and northern) of Italy in the last 10 years.

## Data Availability

Data supporting the conclusions of this article are included within the article and its additional file. The datasets used and analysed during the present study are available from the corresponding author on reasonable request.

## Abbreviations

CanL: Canine leishmaniosis
ZVL: Zoonotic visceral leishmaniosis
EL: Equine leishmaniosis
CI: Confidence interval
IFAT: Immunofluorescence antibody test
m.a.s.l.: Meters above sea level
GLMs: General linear models
AIC: Akaikeʼs information criterion
OR: Odds ratios; SD: standard deviation
