# Supplementary material for: Detecting antibodies to Leishmania infantum in horses from areas with different epizooticity levels of canine leishmaniosis and a retrospective revision of Italian data
Source: Parasit Vectors. 2020 Oct 22;13:530. doi: 10.1186/s13071-020-04385-8 (PMC7583181; doi:10.1186/s13071-020-04385-8)
Supplement: Supplementary file 1 — Additional file 1: Table S1. Comparison of prevalence of L. infantum infection among wildlife and domestic animals from two macro-areas (central and northern) of Italy in the last 10 years. [file 13071_2020_4385_MOESM1_ESM.docx]

# Table S1 - Comparison of prevalence of *L. infantum* infection among wildlife and domestic animals from two macro-areas (central, northern) of Italy in the last 10 years.

| **Location (macro-areas)** | | **Species** | **Percentage of positive (test assayed)** | **Reference** |
| --- | --- | --- | --- | --- |
| **Northern Italy** | Piedmont | Red fox | 12.26% (PCR) | [51] |
|  | Piedmont | Wolf | 25.71% (PCR) | [51] |
|  | Piedmont | European badger | 53.33% (PCR) | [51] |
|  | Piedmont | Dog | 40.35% (Western blotting WB) | [57] |
|  | Northern Italy (region not specified) | Dog | 21.6% (cumulative result obtained by ELISA, IFAT and rapid in-clinic test) | [27] |
|  | Northern Italy (region not specified) | Cat | 1.3% (IFAT)  0.3% (qPCR) | [58] |
|  | Lombardy | Cat | 8.6% (IFAT or RT-PCR) | [61] |
| **Central Italy** | Central Italy (region not specified) | Dog | 29.6% (cumulative result obtained by ELISA, IFAT and rapid in-clinic test) | [27] |
|  | Tuscany and Liguria | Dog | 12% (ELISA) | [62] |
|  | Umbria | Dog | 21.34% (IFAT) | [63] |
|  | Latium and Tuscany | Dog | 2.5% (IFAT) | [64] |
|  | Emilia Romagna | Cat | 11.2% (IFAT) | [65] |
|  | Umbria | Cat | 10.83% (IFAT) | [66] |
|  | Central Italy (region not specified) | Cat | 1.3% (IFAT)  1.3% (qPCR) | [58] |
|  | Abruzzo | Cat | 3% (IFAT) | [67] |
|  | Tuscany | Red fox | 52.2% (PCR) | [50] |
|  | Tuscany | Hare | 9.8% (PCR) | [59] |
|  | Tuscany | Hare | 0.9% (IFAT) | [49] |
|  | Tuscany (Isle of Montecristo) | Black Rat | 15.5% (PCR) | [60] |
